# {4-[(3-Formyl-4-hy­droxy­phen­yl)diazen­yl]benzoato}triphenyl­tin

**DOI:** 10.1107/S160053681002708X

**Published:** 2010-07-14

**Authors:** Smita Basu, Cheerfulman Masharing, Babulal Das

**Affiliations:** a Department of Chemistry, North Eastern Hill University, NEHU Permanent Campus, Umshing, Shillong 793022, India; b Department of Chemistry, Shillong College, Boyce Road, Laitumkhrah, Shillong 793003, India; c Department of Chemistry, Indian Institute of Technology Guwahati, Guwahati 781039, India

## Abstract

In the title compound, [Sn(C_6_H_5_)_3_(C_14_H_9_N_2_O_4_)], the Sn atom has a distorted tetra­hedral geometry with one of the carboxyl­ate O atoms and the C atoms from three phenyl groups. The other carboxyl­ate O atom of the benzoate ligand inter­acts weakly with the Sn atom, with an Sn⋯O distance of 2.790 (2) Å, which causes a distortion of the tetra­hedral coordination geometry.

## Related literature

For related literature on organotin carboxyl­ates, see: Basu Baul *et al.* (1996 ▶, 2004 ▶). For the synthesis, see: Basu Baul *et al.* (2006 ▶).
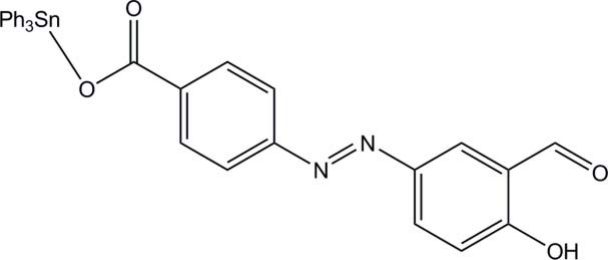


## Experimental

### 

#### Crystal data

[Sn(C_6_H_5_)_3_(C_14_H_9_N_2_O_4_)]
*M* *_r_* = 619.22
Monoclinic, 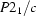

*a* = 8.3751 (2) Å
*b* = 48.8458 (11) Å
*c* = 6.9742 (2) Å
β = 97.262 (1)°
*V* = 2830.18 (12) Å^3^
*Z* = 4
Mo *K*α radiation
μ = 0.94 mm^−1^
*T* = 296 K
0.25 × 0.16 × 0.10 mm

#### Data collection

Bruker SMART APEX CCD area-detector diffractometer
29857 measured reflections
4891 independent reflections
3415 reflections with *I* > 2σ(*I*)
*R*_int_ = 0.059

#### Refinement

*R*[*F* ^2^ > 2σ(*F* ^2^)] = 0.034
*wR*(*F* ^2^) = 0.058
*S* = 0.95
4891 reflections
353 parameters
H-atom parameters constrained
Δρ_max_ = 0.31 e Å^−3^
Δρ_min_ = −0.44 e Å^−3^

### 

Data collection: *SMART* (Bruker, 2001 ▶); cell refinement: *SAINT* (Bruker, 2001 ▶); data reduction: *SAINT*; program(s) used to solve structure: *SHELXS97* (Sheldrick, 2008 ▶); program(s) used to refine structure: *SHELXL97* (Sheldrick, 2008 ▶); molecular graphics: *SHELXTL* (Sheldrick, 2008 ▶); software used to prepare material for publication: *SHELXTL*.

## Supplementary Material

Crystal structure: contains datablocks I, global. DOI: 10.1107/S160053681002708X/is2569sup1.cif

Structure factors: contains datablocks I. DOI: 10.1107/S160053681002708X/is2569Isup2.hkl

Additional supplementary materials:  crystallographic information; 3D view; checkCIF report

## Figures and Tables

**Fig. 1.**
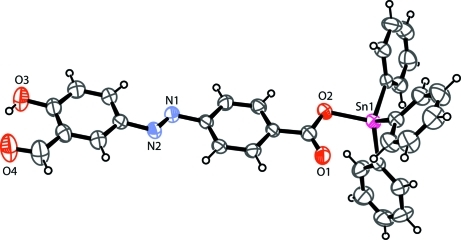
A view of the (1), with the atom-numbering scheme. Displacement ellipsoids are drawn at the 40% probability level and H atoms are shown as small spheres of arbitrary radii.

## supplementary crystallographic information

### Comment

The title compound, (1), was prepared during an ongoing study of the coordination chemistry of organotin carboxylates containing an azo linkage (Basu Baul *et al.*, 1996, 2004). These compounds, especially triphenyltin(IV) complexes, offered interesting structural possibilities. In this context, the crystal structures of many member of this class of compound have been studied. The potential structural usefulness of such systems has prompted in determining the structure of the title compound, (1).

The solid-state structure of complex (1) is a monomeric species with one symmetry-independent molecule in the asymmetric unit where its unit cell contains four molecules (*Z* = 4). The asymmetric unit of the crystal structure contains just one of the principal chemical units (Fig. 1).The primary coordination sphere of the Sn-atom is best described as 4-coordinate with a distorted C3O tetrahedral geometry involving one of the carboxylate O atoms and the C atoms from the three phenyl moieties. The other carboxylate O atom of the benzoate ligand also coordinates weakly to the Sn atom with the Sn1···O1 distance being 2.790 (2) Å. The interaction is the cause of the distortion of the tetrahedral primary coordination sphere, but the Sn···O is considered to be too long for the Sn atom to be described as truly 5-coordinate. In addition, the bond angles around the Sn atom in (I) are more consistent with tetrahedral environment than a trigonal bipyramidal five coordinate environment. If the longer of the Sn1···O1 interaction is interpreted as significant bonding interaction, then the geometry about the tin atom would be described as *cis*-R3SnO2 trigonal bipyramidal with atoms C21, C27, C15 defining the trigonal plane. The unit cell projection of the compound reveals that there is no intermolecular carboxylate bridging. The geometry at the tin atom is intermediate between tetrahedral and *cis*-trigonal bipyramidal, in which the carboxylato ligand spans equatorial and axial sites.

### Experimental

The preparation and spectroscopic data of the title compound have been described by Basu Baul *et al.* (2006).

### Refinement

All H atoms were placed geometrically (C—H = 0.93 and O—H = 0.82 Å) and treated as riding, with *U*_iso_(H) = 1.2*U*_eq_(C) or 1.5*U*_eq_(O).

### Crystal data

**Table:**

[Sn(C_6_H_5_)_3_(C_14_H_9_N_2_O_4_)]	*F*(000) = 1248
*M**_r_* = 619.22	*D*_x_ = 1.453 Mg m^−^^3^
Monoclinic, *P*2_1_/*c*	Mo *K*α radiation, λ = 0.71073 Å
Hall symbol: -P 2ybc	Cell parameters from 9937 reflections
*a* = 8.3751 (2) Å	θ = 0.8–27.3°
*b* = 48.8458 (11) Å	µ = 0.94 mm^−^^1^
*c* = 6.9742 (2) Å	*T* = 296 K
β = 97.262 (1)°	Plates, yellow
*V* = 2830.18 (12) Å^3^	0.25 × 0.16 × 0.10 mm
*Z* = 4	

### Data collection

**Table:**

Bruker SMART APEX CCD area-detector diffractometer	3415 reflections with *I* > 2σ(*I*)
Radiation source: fine-focus sealed tube	*R*_int_ = 0.059
graphite	θ_max_ = 25.0°, θ_min_ = 1.7°
φ and ω scans	*h* = −9→9
29857 measured reflections	*k* = −57→57
4891 independent reflections	*l* = −8→8

### Refinement

**Table:**

Refinement on *F*^2^	Primary atom site location: structure-invariant direct methods
Least-squares matrix: full	Secondary atom site location: difference Fourier map
*R*[*F*^2^ > 2σ(*F*^2^)] = 0.034	Hydrogen site location: inferred from neighbouring sites
*wR*(*F*^2^) = 0.058	H-atom parameters constrained
*S* = 0.95	*w* = 1/[σ^2^(*F*_o_^2^) + (0.023*P*)^2^] where *P* = (*F*_o_^2^ + 2*F*_c_^2^)/3
4891 reflections	(Δ/σ)_max_ = 0.001
353 parameters	Δρ_max_ = 0.31 e Å^−^^3^
0 restraints	Δρ_min_ = −0.44 e Å^−^^3^

### Special details

**Table:**

Geometry. All e.s.d.'s (except the e.s.d. in the dihedral angle between two l.s. planes) are estimated using the full covariance matrix. The cell e.s.d.'s are taken into account individually in the estimation of e.s.d.'s in distances, angles and torsion angles; correlations between e.s.d.'s in cell parameters are only used when they are defined by crystal symmetry. An approximate (isotropic) treatment of cell e.s.d.'s is used for estimating e.s.d.'s involving l.s. planes.
Refinement. Refinement of *F*^2^ against ALL reflections. The weighted *R*-factor *wR* and goodness of fit *S* are based on *F*^2^, conventional *R*-factors *R* are based on *F*, with *F* set to zero for negative *F*^2^. The threshold expression of *F*^2^ > σ(*F*^2^) is used only for calculating *R*-factors(gt) *etc*. and is not relevant to the choice of reflections for refinement. *R*-factors based on *F*^2^ are statistically about twice as large as those based on *F*, and *R*- factors based on ALL data will be even larger.

### Fractional atomic coordinates and isotropic or equivalent isotropic displacement parameters (Å^2^)

**Table:**

	*x*	*y*	*z*	*U*_iso_*/*U*_eq_	
C1	0.6547 (3)	0.11657 (6)	0.9304 (5)	0.0516 (8)	
C2	0.6036 (3)	0.09446 (5)	0.7896 (4)	0.0442 (7)	
C3	0.5061 (3)	0.07333 (6)	0.8418 (4)	0.0516 (8)	
H3A	0.4803	0.0724	0.9674	0.062*	
C4	0.4479 (3)	0.05383 (6)	0.7093 (5)	0.0526 (8)	
H4	0.3820	0.0399	0.7448	0.063*	
C5	0.4873 (3)	0.05497 (6)	0.5236 (5)	0.0463 (8)	
C6	0.5886 (3)	0.07506 (6)	0.4717 (4)	0.0569 (8)	
H6	0.6189	0.0753	0.3480	0.068*	
C7	0.6451 (3)	0.09494 (6)	0.6040 (5)	0.0545 (8)	
H7	0.7116	0.1088	0.5678	0.065*	
C8	0.2627 (4)	0.00218 (6)	0.2606 (5)	0.0519 (8)	
C9	0.1587 (4)	−0.01790 (6)	0.3020 (5)	0.0625 (9)	
H9	0.1308	−0.0194	0.4265	0.075*	
C10	0.0936 (4)	−0.03626 (6)	0.1580 (5)	0.0586 (9)	
C11	0.1364 (4)	−0.03351 (7)	−0.0275 (6)	0.0622 (9)	
C12	0.2416 (4)	−0.01337 (7)	−0.0683 (5)	0.0742 (10)	
H12	0.2702	−0.0118	−0.1924	0.089*	
C13	0.3043 (4)	0.00436 (6)	0.0740 (5)	0.0671 (9)	
H13	0.3753	0.0180	0.0457	0.081*	
C14	−0.0161 (4)	−0.05678 (8)	0.2034 (6)	0.0924 (12)	
H14	−0.0394	−0.0578	0.3300	0.111*	
C15	0.9102 (4)	0.19239 (5)	0.9057 (4)	0.0453 (7)	
C16	1.0741 (4)	0.19735 (6)	0.9357 (4)	0.0615 (9)	
H16	1.1375	0.1881	1.0341	0.074*	
C17	1.1451 (5)	0.21562 (8)	0.8234 (6)	0.0850 (11)	
H17	1.2558	0.2184	0.8452	0.102*	
C18	1.0549 (7)	0.22972 (7)	0.6805 (6)	0.0909 (14)	
H18	1.1036	0.2423	0.6065	0.109*	
C19	0.8933 (6)	0.22542 (7)	0.6457 (5)	0.0817 (11)	
H19	0.8316	0.2350	0.5476	0.098*	
C20	0.8212 (4)	0.20680 (6)	0.7567 (5)	0.0623 (9)	
H20	0.7109	0.2038	0.7312	0.075*	
C21	0.9745 (3)	0.14332 (5)	1.2753 (4)	0.0449 (8)	
C22	0.9802 (4)	0.14661 (6)	1.4718 (5)	0.0633 (9)	
H22	0.9087	0.1586	1.5199	0.076*	
C23	1.0892 (5)	0.13256 (8)	1.5982 (5)	0.0833 (11)	
H23	1.0906	0.1349	1.7307	0.100*	
C24	1.1959 (4)	0.11507 (7)	1.5296 (7)	0.0837 (11)	
H24	1.2694	0.1054	1.6150	0.100*	
C25	1.1942 (4)	0.11190 (7)	1.3350 (7)	0.0903 (12)	
H25	1.2673	0.1002	1.2871	0.108*	
C26	1.0847 (4)	0.12598 (7)	1.2107 (5)	0.0737 (10)	
H26	1.0849	0.1237	1.0783	0.088*	
C27	0.6131 (3)	0.18359 (6)	1.2020 (4)	0.0494 (8)	
C28	0.5985 (4)	0.21167 (7)	1.1858 (4)	0.0658 (9)	
H28	0.6671	0.2213	1.1149	0.079*	
C29	0.4843 (5)	0.22580 (7)	1.2725 (5)	0.0891 (12)	
H29	0.4754	0.2447	1.2585	0.107*	
C30	0.3850 (4)	0.21211 (9)	1.3783 (5)	0.0871 (12)	
H30	0.3084	0.2217	1.4371	0.105*	
C31	0.3965 (4)	0.18456 (9)	1.3989 (5)	0.0833 (11)	
H31	0.3279	0.1753	1.4714	0.100*	
C32	0.5107 (4)	0.17018 (6)	1.3119 (5)	0.0695 (10)	
H32	0.5187	0.1513	1.3275	0.083*	
N1	0.4264 (3)	0.03633 (5)	0.3724 (3)	0.0567 (7)	
N2	0.3234 (3)	0.01978 (5)	0.4159 (4)	0.0568 (7)	
O1	0.6276 (3)	0.11589 (4)	1.0984 (3)	0.0693 (6)	
O2	0.7321 (2)	0.13706 (4)	0.8634 (3)	0.0578 (6)	
O3	0.0779 (3)	−0.05032 (5)	−0.1753 (3)	0.0941 (8)	
H3	0.0189	−0.0619	−0.1356	0.141*	
O4	−0.0820 (3)	−0.07322 (5)	0.0854 (4)	0.1118 (10)	
Sn1	0.80328 (2)	0.164169 (4)	1.08190 (3)	0.04767 (10)	

### Atomic displacement parameters (Å^2^)

**Table:**

	*U*^11^	*U*^22^	*U*^33^	*U*^12^	*U*^13^	*U*^23^
C1	0.044 (2)	0.050 (2)	0.059 (3)	0.0018 (16)	−0.0031 (19)	0.002 (2)
C2	0.046 (2)	0.0366 (19)	0.049 (2)	−0.0018 (15)	0.0014 (16)	0.0044 (16)
C3	0.057 (2)	0.055 (2)	0.042 (2)	−0.0031 (17)	0.0033 (16)	0.0057 (17)
C4	0.058 (2)	0.046 (2)	0.052 (2)	−0.0107 (16)	0.0021 (18)	0.0089 (17)
C5	0.048 (2)	0.0394 (19)	0.050 (2)	−0.0012 (15)	−0.0026 (17)	0.0036 (17)
C6	0.065 (2)	0.059 (2)	0.047 (2)	−0.0092 (19)	0.0096 (17)	0.0032 (18)
C7	0.054 (2)	0.049 (2)	0.059 (2)	−0.0102 (16)	0.0058 (18)	0.0061 (18)
C8	0.057 (2)	0.042 (2)	0.055 (2)	−0.0002 (17)	−0.0016 (19)	0.0031 (17)
C9	0.059 (2)	0.058 (2)	0.070 (3)	−0.0023 (19)	0.0064 (19)	−0.005 (2)
C10	0.050 (2)	0.050 (2)	0.076 (3)	−0.0053 (17)	0.008 (2)	−0.003 (2)
C11	0.058 (2)	0.050 (2)	0.076 (3)	−0.0007 (18)	−0.002 (2)	−0.007 (2)
C12	0.089 (3)	0.071 (3)	0.061 (3)	−0.011 (2)	0.008 (2)	0.001 (2)
C13	0.082 (3)	0.055 (2)	0.062 (3)	−0.0125 (19)	0.001 (2)	0.001 (2)
C14	0.090 (3)	0.087 (3)	0.104 (3)	−0.022 (2)	0.026 (3)	−0.025 (3)
C15	0.052 (2)	0.0361 (18)	0.048 (2)	−0.0001 (16)	0.0041 (17)	−0.0045 (15)
C16	0.059 (2)	0.063 (2)	0.063 (2)	−0.0098 (19)	0.0104 (19)	0.0009 (18)
C17	0.084 (3)	0.080 (3)	0.098 (3)	−0.029 (2)	0.037 (3)	−0.006 (2)
C18	0.138 (4)	0.060 (3)	0.088 (3)	−0.009 (3)	0.065 (3)	0.007 (2)
C19	0.125 (4)	0.061 (3)	0.064 (3)	0.025 (3)	0.030 (3)	0.020 (2)
C20	0.071 (2)	0.052 (2)	0.063 (2)	0.0065 (19)	0.006 (2)	0.0019 (19)
C21	0.049 (2)	0.0357 (18)	0.050 (2)	0.0020 (15)	0.0092 (17)	0.0011 (15)
C22	0.058 (2)	0.073 (2)	0.060 (3)	0.0152 (19)	0.014 (2)	0.000 (2)
C23	0.092 (3)	0.106 (3)	0.050 (3)	0.017 (3)	0.001 (2)	0.013 (2)
C24	0.077 (3)	0.075 (3)	0.093 (4)	0.013 (2)	−0.014 (3)	0.022 (2)
C25	0.093 (3)	0.084 (3)	0.092 (3)	0.039 (2)	0.001 (3)	−0.002 (3)
C26	0.086 (3)	0.078 (3)	0.055 (2)	0.029 (2)	0.000 (2)	−0.007 (2)
C27	0.042 (2)	0.049 (2)	0.056 (2)	−0.0015 (16)	0.0061 (16)	0.0010 (16)
C28	0.067 (2)	0.057 (2)	0.076 (3)	0.0048 (19)	0.0214 (19)	0.0021 (19)
C29	0.099 (3)	0.064 (3)	0.110 (3)	0.025 (2)	0.038 (3)	−0.004 (2)
C30	0.068 (3)	0.100 (3)	0.098 (3)	0.022 (3)	0.027 (2)	−0.011 (3)
C31	0.069 (3)	0.089 (3)	0.100 (3)	−0.007 (2)	0.041 (2)	−0.001 (2)
C32	0.064 (2)	0.059 (2)	0.089 (3)	−0.0028 (19)	0.025 (2)	0.0030 (19)
N1	0.0621 (19)	0.0483 (17)	0.0575 (19)	−0.0017 (14)	−0.0014 (15)	−0.0015 (14)
N2	0.0601 (19)	0.0444 (17)	0.063 (2)	−0.0096 (14)	−0.0039 (15)	−0.0016 (14)
O1	0.0905 (17)	0.0604 (14)	0.0577 (16)	−0.0191 (12)	0.0120 (14)	−0.0072 (12)
O2	0.0657 (14)	0.0455 (13)	0.0619 (14)	−0.0161 (11)	0.0072 (11)	0.0026 (10)
O3	0.104 (2)	0.0837 (19)	0.0918 (19)	−0.0214 (15)	0.0032 (15)	−0.0350 (16)
O4	0.114 (2)	0.100 (2)	0.124 (2)	−0.0463 (17)	0.0239 (18)	−0.0431 (18)
Sn1	0.04686 (15)	0.04285 (14)	0.05363 (16)	−0.00166 (11)	0.00767 (10)	0.00238 (11)

### Geometric parameters (Å, °)

**Table:**

C1—O1	1.222 (3)	C17—H17	0.9300
C1—O2	1.310 (3)	C18—C19	1.360 (4)
C1—C2	1.486 (4)	C18—H18	0.9300
C2—C7	1.382 (3)	C19—C20	1.382 (4)
C2—C3	1.393 (3)	C19—H19	0.9300
C3—C4	1.373 (3)	C20—H20	0.9300
C3—H3A	0.9300	C21—C26	1.370 (4)
C4—C5	1.377 (4)	C21—C22	1.374 (4)
C4—H4	0.9300	C21—Sn1	2.104 (3)
C5—C6	1.375 (3)	C22—C23	1.371 (4)
C5—N1	1.437 (3)	C22—H22	0.9300
C6—C7	1.381 (3)	C23—C24	1.366 (4)
C6—H6	0.9300	C23—H23	0.9300
C7—H7	0.9300	C24—C25	1.364 (4)
C8—C9	1.367 (4)	C24—H24	0.9300
C8—C13	1.393 (4)	C25—C26	1.366 (4)
C8—N2	1.425 (3)	C25—H25	0.9300
C9—C10	1.404 (4)	C26—H26	0.9300
C9—H9	0.9300	C27—C28	1.381 (3)
C10—C11	1.392 (4)	C27—C32	1.384 (4)
C10—C14	1.422 (4)	C27—Sn1	2.116 (3)
C11—O3	1.360 (3)	C28—C29	1.380 (4)
C11—C12	1.374 (4)	C28—H28	0.9300
C12—C13	1.371 (4)	C29—C30	1.355 (4)
C12—H12	0.9300	C29—H29	0.9300
C13—H13	0.9300	C30—C31	1.356 (4)
C14—O4	1.230 (4)	C30—H30	0.9300
C14—H14	0.9300	C31—C32	1.387 (4)
C15—C16	1.384 (4)	C31—H31	0.9300
C15—C20	1.391 (4)	C32—H32	0.9300
C15—Sn1	2.119 (3)	N1—N2	1.248 (3)
C16—C17	1.371 (4)	O2—Sn1	2.0498 (18)
C16—H16	0.9300	O3—H3	0.8200
C17—C18	1.360 (5)		
			
O1—C1—O2	121.5 (3)	C19—C18—H18	120.0
O1—C1—C2	122.8 (3)	C18—C19—C20	119.8 (4)
O2—C1—C2	115.7 (3)	C18—C19—H19	120.1
C7—C2—C3	118.8 (3)	C20—C19—H19	120.1
C7—C2—C1	121.4 (3)	C19—C20—C15	121.4 (3)
C3—C2—C1	119.7 (3)	C19—C20—H20	119.3
C4—C3—C2	120.6 (3)	C15—C20—H20	119.3
C4—C3—H3A	119.7	C26—C21—C22	117.4 (3)
C2—C3—H3A	119.7	C26—C21—Sn1	121.4 (2)
C3—C4—C5	119.8 (3)	C22—C21—Sn1	121.2 (2)
C3—C4—H4	120.1	C23—C22—C21	121.3 (3)
C5—C4—H4	120.1	C23—C22—H22	119.4
C6—C5—C4	120.3 (3)	C21—C22—H22	119.4
C6—C5—N1	115.6 (3)	C24—C23—C22	120.0 (3)
C4—C5—N1	124.0 (3)	C24—C23—H23	120.0
C5—C6—C7	119.8 (3)	C22—C23—H23	120.0
C5—C6—H6	120.1	C25—C24—C23	119.6 (3)
C7—C6—H6	120.1	C25—C24—H24	120.2
C6—C7—C2	120.5 (3)	C23—C24—H24	120.2
C6—C7—H7	119.7	C24—C25—C26	119.7 (4)
C2—C7—H7	119.7	C24—C25—H25	120.1
C9—C8—C13	119.6 (3)	C26—C25—H25	120.1
C9—C8—N2	116.6 (3)	C25—C26—C21	121.9 (3)
C13—C8—N2	123.8 (3)	C25—C26—H26	119.0
C8—C9—C10	120.5 (3)	C21—C26—H26	119.0
C8—C9—H9	119.8	C28—C27—C32	117.4 (3)
C10—C9—H9	119.8	C28—C27—Sn1	118.4 (2)
C11—C10—C9	118.8 (3)	C32—C27—Sn1	123.8 (2)
C11—C10—C14	121.6 (3)	C29—C28—C27	121.3 (3)
C9—C10—C14	119.6 (3)	C29—C28—H28	119.3
O3—C11—C12	117.0 (3)	C27—C28—H28	119.3
O3—C11—C10	122.4 (3)	C30—C29—C28	120.0 (3)
C12—C11—C10	120.6 (3)	C30—C29—H29	120.0
C13—C12—C11	119.9 (3)	C28—C29—H29	120.0
C13—C12—H12	120.0	C29—C30—C31	120.4 (3)
C11—C12—H12	120.0	C29—C30—H30	119.8
C12—C13—C8	120.7 (3)	C31—C30—H30	119.8
C12—C13—H13	119.7	C30—C31—C32	120.0 (3)
C8—C13—H13	119.7	C30—C31—H31	120.0
O4—C14—C10	124.0 (4)	C32—C31—H31	120.0
O4—C14—H14	118.0	C27—C32—C31	120.8 (3)
C10—C14—H14	118.0	C27—C32—H32	119.6
C16—C15—C20	116.9 (3)	C31—C32—H32	119.6
C16—C15—Sn1	120.7 (2)	N2—N1—C5	115.1 (2)
C20—C15—Sn1	122.5 (2)	N1—N2—C8	113.4 (3)
C17—C16—C15	121.4 (3)	C1—O2—Sn1	109.81 (19)
C17—C16—H16	119.3	C11—O3—H3	109.5
C15—C16—H16	119.3	O2—Sn1—C21	105.94 (9)
C18—C17—C16	120.5 (4)	O2—Sn1—C27	114.91 (9)
C18—C17—H17	119.8	C21—Sn1—C27	116.72 (11)
C16—C17—H17	119.8	O2—Sn1—C15	95.37 (9)
C17—C18—C19	120.0 (4)	C21—Sn1—C15	112.52 (12)
C17—C18—H18	120.0	C27—Sn1—C15	109.42 (11)
			
O1—C1—C2—C7	−175.7 (3)	C23—C24—C25—C26	−0.7 (6)
O2—C1—C2—C7	4.0 (4)	C24—C25—C26—C21	−0.3 (5)
O1—C1—C2—C3	7.1 (4)	C22—C21—C26—C25	1.5 (5)
O2—C1—C2—C3	−173.2 (2)	Sn1—C21—C26—C25	−178.6 (3)
C7—C2—C3—C4	−2.1 (4)	C32—C27—C28—C29	−1.2 (5)
C1—C2—C3—C4	175.2 (3)	Sn1—C27—C28—C29	−175.4 (2)
C2—C3—C4—C5	0.6 (4)	C27—C28—C29—C30	0.9 (5)
C3—C4—C5—C6	2.0 (4)	C28—C29—C30—C31	−0.3 (6)
C3—C4—C5—N1	−177.0 (2)	C29—C30—C31—C32	0.1 (6)
C4—C5—C6—C7	−3.0 (4)	C28—C27—C32—C31	1.1 (5)
N1—C5—C6—C7	176.0 (2)	Sn1—C27—C32—C31	174.9 (2)
C5—C6—C7—C2	1.5 (4)	C30—C31—C32—C27	−0.6 (5)
C3—C2—C7—C6	1.1 (4)	C6—C5—N1—N2	−173.7 (2)
C1—C2—C7—C6	−176.2 (3)	C4—C5—N1—N2	5.3 (4)
C13—C8—C9—C10	0.0 (4)	C5—N1—N2—C8	178.3 (2)
N2—C8—C9—C10	−179.7 (3)	C9—C8—N2—N1	175.0 (2)
C8—C9—C10—C11	−0.4 (4)	C13—C8—N2—N1	−4.6 (4)
C8—C9—C10—C14	−179.2 (3)	O1—C1—O2—Sn1	3.0 (3)
C9—C10—C11—O3	−179.5 (3)	C2—C1—O2—Sn1	−176.74 (18)
C14—C10—C11—O3	−0.8 (5)	C1—O2—Sn1—C21	66.29 (19)
C9—C10—C11—C12	0.7 (5)	C1—O2—Sn1—C27	−64.1 (2)
C14—C10—C11—C12	179.4 (3)	C1—O2—Sn1—C15	−178.44 (19)
O3—C11—C12—C13	179.7 (3)	C26—C21—Sn1—O2	38.5 (2)
C10—C11—C12—C13	−0.5 (5)	C22—C21—Sn1—O2	−141.6 (2)
C11—C12—C13—C8	0.1 (5)	C26—C21—Sn1—C27	167.8 (2)
C9—C8—C13—C12	0.2 (5)	C22—C21—Sn1—C27	−12.2 (3)
N2—C8—C13—C12	179.8 (3)	C26—C21—Sn1—C15	−64.5 (3)
C11—C10—C14—O4	−0.7 (6)	C22—C21—Sn1—C15	115.5 (2)
C9—C10—C14—O4	178.0 (3)	C28—C27—Sn1—O2	−117.3 (2)
C20—C15—C16—C17	−0.1 (4)	C32—C27—Sn1—O2	68.9 (3)
Sn1—C15—C16—C17	−179.8 (2)	C28—C27—Sn1—C21	117.7 (2)
C15—C16—C17—C18	1.0 (5)	C32—C27—Sn1—C21	−56.0 (3)
C16—C17—C18—C19	−1.2 (5)	C28—C27—Sn1—C15	−11.5 (3)
C17—C18—C19—C20	0.3 (5)	C32—C27—Sn1—C15	174.8 (2)
C18—C19—C20—C15	0.6 (5)	C16—C15—Sn1—O2	−115.4 (2)
C16—C15—C20—C19	−0.8 (4)	C20—C15—Sn1—O2	64.9 (2)
Sn1—C15—C20—C19	179.0 (2)	C16—C15—Sn1—C21	−5.7 (3)
C26—C21—C22—C23	−1.6 (5)	C20—C15—Sn1—C21	174.6 (2)
Sn1—C21—C22—C23	178.5 (2)	C16—C15—Sn1—C27	125.8 (2)
C21—C22—C23—C24	0.6 (5)	C20—C15—Sn1—C27	−53.9 (2)
C22—C23—C24—C25	0.6 (5)		
